# Supplementary material for: Factors influencing the use of therapeutic footwear in persons with diabetes mellitus and loss of protective sensation: A focus group study
Source: PLoS One. 2023 Jan 12;18(1):e0280264. doi: 10.1371/journal.pone.0280264 (PMC9836263; doi:10.1371/journal.pone.0280264)
Supplement: S2 File — (PDF) [file pone.0280264.s002.pdf]

S2. Appendix Interview guide for the focus group session of group 1

Introduction (15 min)

- Intro of the moderator & researchers & explaining the rules of the session
- Introduction of the participants (name, diabetes type, use of therapeutic footwear)

Knowledge (5 minutes)

A lot of patients with DM get therapeutic footwear prescribed to them.

1. Do have a general idea what could be the most important reason is for receiving therapeutic footwear?
2. Do you know what type of therapeutic footwear are available?

After this round, the assistant moderator gave a 5 min presentation on the different types of therapeutic footwear and when this footwear is used

Context (15 minutes)

1. Who has already thought about the chance of getting therapeutic footwear prescribed, in the future? What are your thoughts on that?
2. Imagine for a moment that you are getting therapeutic footwear prescribed, what questions pop up? What are your expectations/what kind of feeling do you get by this idea and where does this feeling come from? Would this affect the use of therapeutic footwear?
3. Do you know people who are using therapeutic footwear? Did you ever discuss this with them?
4. What should we take according to you into consideration if we had to develop new therapeutic footwear? Are there additional factors that were not discussed yet but are important to consider?
5. What is your ideal image of a perfect therapeutic footwear?

Orientation (10 minutes)

1. If you would hear you had to use therapeutic footwear, would you orientate? If so, what would you pay attention on?
2. Which sources would you use in order to gather information?
3. Which therapeutic footwear suppliers do you already know?

Decision, fitting and purchase (15 minutes)

1. After a physician has prescribed therapeutic footwear, which steps follow next, according to you?
2. One has to make a choice for the footwear you will get, what should be your role in this process? What do you think is important to share with the orthopedic shoe technician? What should happen during the moment where therapeutic footwear is chosen and what should be absolutely avoided?
3. Therapeutic footwear are fitted by an orthopedic shoe technician. This male or female takes the measurements of your foot and also takes into consideration the physician's prescription, in order to get shoes that help medical wise in preventing foot problems from happening. Imagine you are in the fitting room of an orthopedic shoe technician, what questions would you ask? What do you think is important for him/her to know about certain preferences of yours?
4. When would you use therapeutic footwear?

Use (10 minutes)

1. It is important that therapeutic footwear are used during the whole day. How long do you use your non-therapeutic footwear per day? Would that differ for therapeutic footwear? What would help to use therapeutic footwear for a longer period of time?
2. During what activities would you use therapeutic footwear and for what reasons?
3. During what activities would you not use therapeutic footwear and for what reasons?

Appearance (15 minutes)

1. *(Prototype shoes are shown by the assistant moderator)* What do you think about these shoes?
2. *(Purpose and function of the prototype shoes are given by the assistant moderator)* If the shoes stay looking like this but they function properly, would you use the shoes?
3. Is the opinion of others important for the use of therapeutic footwear?

Break of 10 minutes

Wind-up (25 min)

1. *(A list with the factors that could influence the use of therapeutic footwear that were mentioned during the discussion was shown by the assistant moderator and the Master student)* Are there still some factors that you find important that we are missing on the list?
2. Could you rank (in a poll, 3x) what factor is number 1, 2 and 3? Could you also give the reasoning for your ranking?
