# Supplementary material for: Factors influencing the use of therapeutic footwear in persons with diabetes mellitus and loss of protective sensation: A focus group study
Source: PLoS One. 2023 Jan 12;18(1):e0280264. doi: 10.1371/journal.pone.0280264 (PMC9836263; doi:10.1371/journal.pone.0280264)
Supplement: S3 File — (PDF) [file pone.0280264.s003.pdf]

S3. Appendix Interview guide for the focus group sessions of group 2 & 3

Introduction (15 min)

- Intro of the moderator & researchers & explaining the rules of the session
- Introduction of the participants (name, diabetes type, use of therapeutic footwear)

Knowledge (10 minutes)

A lot of patients with DM get therapeutic footwear prescribed to them.

1. Do you know why therapeutic footwear are prescribed to patients with DM?
2. Do you know what type of therapeutic footwear are available?

After this round, the assistant moderator gave a 5 min presentation on the different types of therapeutic footwear and when this footwear is used

Experience (15-20 minutes)

1. When/during what activities do you use your therapeutic footwear and why? How many hours per day and how many days per week do you use therapeutic footwear and why? When do you not use them and why?
2. Do you have issues with keeping your balance? Does this change when you use therapeutic footwear?
3. What is the most important reason for you to use therapeutic footwear?
4. Could you share your positive experiences? *(similar experience is askes around in the group by the moderator)*
5. Could you share your negative experiences? *(similar experience is askes around in the group by the moderator)*

Wishes (15 minutes)

1. What should we take according to you into consideration if we had to develop new therapeutic footwear?
2. Are there additional factors that were not discussed yet but are important to consider?
3. What is your ideal image of a perfect therapeutic footwear?

Effectiveness & usability (15-20 minutes)

Using therapeutic footwear effects the walking quality, pain, wounds/ulcers, sprain and balance for instance. There are a lot of other effects ofcourse.

1. What changes did you experience after using therapeutic footwear?
2. What effects of therapeutic footwear are important and why?
3. Does this influence you in using therapeutic footwear?
4. Are there other effects that influence the use of therapeutic footwear?

There are a lot of practical matters around using therapeutic footwear, for instance the donning & doffing of therapeutic footwear, the maintenance, the weight that you experience, the fit or material?

1. What factors related to the usability of therapeutic footwear are the most important and why?
2. Does that influence the use of therapeutic footwear?
3. Are there other factors related to the usability that also influence the use of therapeutic footwear.

Appearance (20 minutes)

1. How would you assess the appearance of your current therapeutic footwear? Does that influence the use of therapeutic footwear?
2. *(Prototype shoes are shown and purpose & function are explained by the assistant moderator)* What do you think about the appearance and functional traits of these shoes?
3. Would you, based on the appearance and/or functional traits of the shoes, use them or not and why?
4. Is the opinion of others important for the use of therapeutic footwear?
5. Were you involved in the choice of your own therapeutic footwear and does the extent of your involvement influence the use of therapeutic footwear?

Break of 10 minutes

Wind-up (20-25 min)

1. *(A list with the factors that could influence the use of therapeutic footwear that were mentioned during the discussion was shown by the assistant moderator and the Master student)* Are there still some factors that you find important that we are missing on the list?
2. What are the three most important factors for you? *(the factors are shown in poll).*
