# Supplementary figures and images for: Factors influencing the use of therapeutic footwear in persons with diabetes mellitus and loss of protective sensation: A focus group study
Source: PLoS One. 2023 Jan 12;18(1):e0280264. doi: 10.1371/journal.pone.0280264 (PMC9836263; doi:10.1371/journal.pone.0280264)

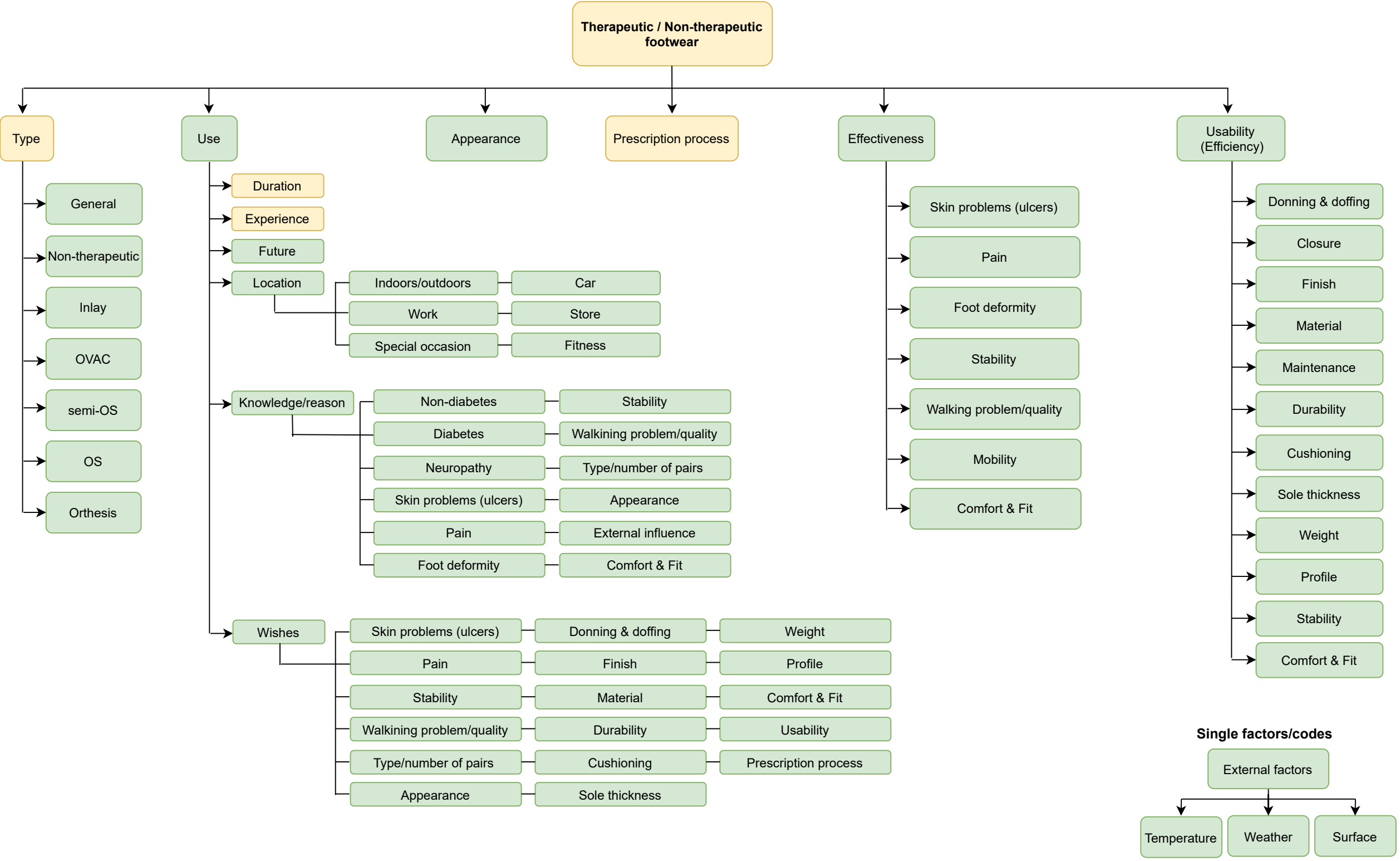

Supplement: S4 File — (PDF) [file pone.0280264.s004.pdf]

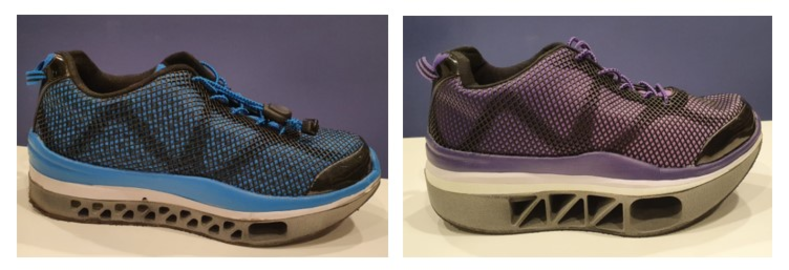

Supplement: S1 Fig — (TIF) [file pone.0280264.s005.tif]
