# Supplementary material for: Factors influencing the use of therapeutic footwear in persons with diabetes mellitus and loss of protective sensation: A focus group study
Source: PLoS One. 2023 Jan 12;18(1):e0280264. doi: 10.1371/journal.pone.0280264 (PMC9836263; doi:10.1371/journal.pone.0280264)
Supplement: S1 Table — (PDF) [file pone.0280264.s006.pdf]

S1 Table. Non-diabetes related comorbidities per focus group.

Characteristics	Group-noTF&noHoU n=8	Group-TF&noHoU n=8	Group-TF&HoU n=8
Comorbidities (non-diabetes related)			
Hypertension	4	4	4
Arthrosis (back/hip/knee/wrists)	2	5	1
Heart failure/arrhythmia	0	3	3
Sleep apnea	1	2	1
Drop foot	0	1	1
Asthma	2	0	0
Bechterew	0	0	1
Hernia	1	0	0
Neuralgic amyotrophy	0	1	0
Incomplete spinal cord injury (L4 and L5)	0	1	0
Inclusion body myositis	0	0	1
Crohn’s disease	0	1	0
Early renal insufficiency	0	1	0
Hypothyroidism	0	1	0
