# Supplementary material for: Factors influencing the use of therapeutic footwear in persons with diabetes mellitus and loss of protective sensation: A focus group study
Source: PLoS One. 2023 Jan 12;18(1):e0280264. doi: 10.1371/journal.pone.0280264 (PMC9836263; doi:10.1371/journal.pone.0280264)
Supplement: Attachment — Submitted filename: Reviewer notes.docx [file pone.0280264.s007.docx]

Factors influencing the use of therapeutic footwear in patients with diabetes mellitus and loss of protective sensation.

The aim of the present study was to provide insight in the factors that are perceived important in the use of TF for different groups of patients with DM and DPN. To this aim the authors evaluated three different groups, a) patients without therapeutic footwear (TF) and no history of DFU, b) patients without TF and history of DFU and c) patients with TF and DFU.

The study is nicely conducted and the results are nicely presented, despite that, some changes must be made before publication:

INTRODUCTION:

- To long introduction, the authors must shorten it before publication.

STUDY DESIGN AND DATA ALLOCATION

- The authors state in lines 149-151 that it was not possible due to Covid-19 pandemic to assess physical measurements, it could be a bias in the selection of inclusion and exclusion criteria, it must be discussed as a limitation.

- LOPS: authors declare that patients with LOPS were included in the research, but auto perception of LOPS is not a valid method to diagnose DPN and LOPS, authors must explain it in depth and if no neurological assessment was performed, authors might explain how it can be done and if no possible add as a limitation in the discussion section accordingly.

-

RESULTS:

- Higher risk of ulcer occurrence patients seem to have higher knowledge about DFU complications derived from the results presented in table 2, despite this, the qualitative analyses of the results do not let us to reach any conclusion.

- Also, the three studied groups were well balanced in terms of baseline characteristics. That said, the plausibility of the results may be limited by the relatively low number of participants. Was a sample size calculation performed? 8 patients per groups is very small, this need to be included as a limitation of the study.

- The paper has a big weakness due to the lack of quantitative data, for further research, authors should confirm this results in a quantitative study.

DISCUSSION:

- The discussion is coherent, but I recommend shortening it.
