# Supplementary material for: Factors influencing the use of therapeutic footwear in persons with diabetes mellitus and loss of protective sensation: A focus group study
Source: PLoS One. 2023 Jan 12;18(1):e0280264. doi: 10.1371/journal.pone.0280264 (PMC9836263; doi:10.1371/journal.pone.0280264)
Supplement: Attachment — Submitted filename: Reviewer Comments_ Sayed Ahmed.docx [file pone.0280264.s008.docx]

Reviewer #2 Comment”

Page 3, Line 56, Please check the space between the reference and the following lines. Also, the spacing is inconsistent in other areas, such as Page 3, line 63 and other references on this page and also throughout the article. Please make them all consistent.

Page 4, Line 101. Please check grammar for “proofed”.

Page 5, Line 123. Please clarify if the Orthopedic shoe technicians are titled as “Pedorthists” in the Netherlands. If so, please use the appropriate professional title and please check other studies from the Netherlands have been using the term.

Ref: <https://onlinelibrary.wiley.com/doi/full/10.1002/dmrr.3237>

Page 6, Line 150, Please clarify if there was an option to collect or verify the patient data such as LOPS, and foot deformities from a verified medical record source or referral source other than the patient’s self-report on them.

Page 11, Line 242. Please be consistent with using Capital for “ Appearance” and “prescription process”.
