# Supplementary material for: Factors influencing the use of therapeutic footwear in persons with diabetes mellitus and loss of protective sensation: A focus group study
Source: PLoS One. 2023 Jan 12;18(1):e0280264. doi: 10.1371/journal.pone.0280264 (PMC9836263; doi:10.1371/journal.pone.0280264)
Supplement: Attachment — Submitted filename: Response to reviewers.docx [file pone.0280264.s009.docx]

Dear Editor,

We thank you for considering to publish this article in the PLOS ONE Journal. We also thank you and the reviewers for your time and critical view on the article. We have made a point-by-point response in the table below with our answer to your and the reviewers question or suggestion and which changes were made accordingly in the manuscript.

|  | **Journal requirements** | | | | |
| --- | --- | --- | --- | --- | --- |
| **Nr** | **Editor comments** | | **Authors’ response** | **Change in manuscript** | |
|  | **Comments** | | | | |
| 1 | **Please ensure that your manuscript meets PLOS ONE's style requirements, including those for file naming. The PLOS ONE style templates can be found at**  **https://journals.plos.org/plosone/s/file?id=wjVg/PLOSOne_formatting_sample_main_body.pdf and**  [**https://journals.plos.org/plosone/s/file?id=ba62/PLOSOne_formatting_sample_title_authors_affiliations.pdf**](https://eur03.safelinks.protection.outlook.com/?url=https%3A%2F%2Fjournals.plos.org%2Fplosone%2Fs%2Ffile%3Fid%3Dba62%2FPLOSOne_formatting_sample_title_authors_affiliations.pdf&data=05%7C01%7Ca.malki%40umcg.nl%7C5eeb2baff5fd4dd9a0b708da90274f5b%7C335122f9d4f44d67a2fccd6dc20dde70%7C0%7C0%7C637980797572420627%7CUnknown%7CTWFpbGZsb3d8eyJWIjoiMC4wLjAwMDAiLCJQIjoiV2luMzIiLCJBTiI6Ik1haWwiLCJXVCI6Mn0%3D%7C3000%7C%7C%7C&sdata=b%2B%2BDZlXimaiFi0G9kSwQe2eV3SX4r810f%2Fi%2Bbe581Rg%3D&reserved=0)**.** | | We have checked the PLOS ONE style requirements again and adapted the manuscript, title page and file naming accordingly. | Page 1: new style changes of the ‘title’, ‘authors’ and ‘affiliations’.  Lines 4-6: Title moved to line 4 and removed the ‘bold’ style  Line 9: Author names are not given in ‘bold’ anymore and added some space in between ‘title’ and ‘authors’ as given in [https://journals.plos.org/plosone/s/file?id=ba62/PLOSOne_formatting_sample_title_authors_affiliations.pdf](https://eur03.safelinks.protection.outlook.com/?url=https%3A%2F%2Fjournals.plos.org%2Fplosone%2Fs%2Ffile%3Fid%3Dba62%2FPLOSOne_formatting_sample_title_authors_affiliations.pdf&data=05%7C01%7Ca.malki%40umcg.nl%7C5eeb2baff5fd4dd9a0b708da90274f5b%7C335122f9d4f44d67a2fccd6dc20dde70%7C0%7C0%7C637980797572420627%7CUnknown%7CTWFpbGZsb3d8eyJWIjoiMC4wLjAwMDAiLCJQIjoiV2luMzIiLCJBTiI6Ik1haWwiLCJXVCI6Mn0%3D%7C3000%7C%7C%7C&sdata=b%2B%2BDZlXimaiFi0G9kSwQe2eV3SX4r810f%2Fi%2Bbe581Rg%3D&reserved=0)  Lines 13-17: Added the ‘state’ to the affiliation line. Also added some space in between the author names and the affiliations as given in [https://journals.plos.org/plosone/s/file?id=ba62/PLOSOne_formatting_sample_title_authors_affiliations.pdf](https://eur03.safelinks.protection.outlook.com/?url=https%3A%2F%2Fjournals.plos.org%2Fplosone%2Fs%2Ffile%3Fid%3Dba62%2FPLOSOne_formatting_sample_title_authors_affiliations.pdf&data=05%7C01%7Ca.malki%40umcg.nl%7C5eeb2baff5fd4dd9a0b708da90274f5b%7C335122f9d4f44d67a2fccd6dc20dde70%7C0%7C0%7C637980797572420627%7CUnknown%7CTWFpbGZsb3d8eyJWIjoiMC4wLjAwMDAiLCJQIjoiV2luMzIiLCJBTiI6Ik1haWwiLCJXVCI6Mn0%3D%7C3000%7C%7C%7C&sdata=b%2B%2BDZlXimaiFi0G9kSwQe2eV3SX4r810f%2Fi%2Bbe581Rg%3D&reserved=0).  Lines 20-21: Added the name of the corresponding author and the word ‘Email’ in line 21.  Page 35: supporting file names are changed according to <https://journals.plos.org/plosone/s/file?id=wjVg/PLOSOne_formatting_sample_main_body.pdf>  Supporting file ‘S5_Fig.tif’ is changed to ‘S1_Fig.tif’ | |
| 2 | **We suggest you thoroughly copyedit your manuscript for language usage, spelling, and grammar. If you do not know anyone who can help you do this, you may wish to consider employing a professional scientific editing service.**  **Whilst you may use any professional scientific editing service of your choice, PLOS has partnered with both American Journal Experts (AJE) and Editage to provide discounted services to PLOS authors. Both organizations have experience helping authors meet PLOS guidelines and can provide language editing, translation, manuscript formatting, and figure formatting to ensure your manuscript meets our submission guidelines. To take advantage of our partnership with AJE, visit the AJE website (http://learn.aje.com/plos/) for a 15% discount off AJE services. To take advantage of our partnership with Editage, visit the Editage website (www.editage.com) and enter referral code PLOSEDIT for a 15% discount off Editage services.  If the PLOS editorial team finds any language issues in text that either AJE or Editage has edited, the service provider will re-edit the text for free.**  **Upon resubmission, please provide the following:**  **The name of the colleague or the details of the professional service that edited your manuscript**  **A copy of your manuscript showing your changes by either highlighting them or using track changes (uploaded as a *supporting information* file)**  **A clean copy of the edited manuscript (uploaded as the new *manuscript* file).** | | Thank you for your suggestions. In order to follow your advice to thoroughly edit our manuscript for language usage (both spelling and grammar) we contracted Mrs. Sonja Hintzen, who is a very experienced professional in language editing, and who went through the manuscript and corrected for languages usage, spelling and grammar. |  | |
| 3 | **We note that the grant information you provided in the ‘Funding Information’ and ‘Financial Disclosure’ sections do not match. When you resubmit, please ensure that you provide the correct grant numbers for the awards you received for your study in the ‘Funding Information’ section.** | | Thank you for pointing out this omission to us: we will make sure to match the grant information in both sections upon resubmission. | The following information will be added in both sections:  JMH received the funding for this work by EIT Health: https://eithealth.eu/. The Grant  number was 211018. This work was part of the research project titled: ‘IndiRock'nSole’. The funder had no role in the study design, data-collection and analysis, decision to publish, or preparation of the manuscript. | |
| 4 | **In your Data Availability statement, you have not specified where the minimal data set underlying the results described in your manuscript can be found. PLOS defines a study's minimal data set as the underlying data used to reach the conclusions drawn in the manuscript and any additional data required to replicate the reported study findings in their entirety. All PLOS journals require that the minimal data set be made fully available. For more information about our data policy, please see http://journals.plos.org/plosone/s/data-availability.**  **Upon re-submitting your revised manuscript, please upload your study’s minimal underlying data set as either Supporting Information files or to a stable, public repository and include the relevant URLs, DOIs, or accession numbers within your revised cover letter. For a list of acceptable repositories, please see http://journals.plos.org/plosone/s/data-availability#loc-recommended-repositories. Any potentially identifying patient information must be fully anonymized.**  **Important: If there are ethical or legal restrictions to sharing your data publicly, please explain these restrictions in detail. Please see our guidelines for more information on what we consider unacceptable restrictions to publicly sharing data:** [**http://journals.plos.org/plosone/s/data-availability#loc**](https://eur03.safelinks.protection.outlook.com/?url=http%3A%2F%2Fjournals.plos.org%2Fplosone%2Fs%2Fdata-availability%23loc&data=05%7C01%7Ca.malki%40umcg.nl%7C5eeb2baff5fd4dd9a0b708da90274f5b%7C335122f9d4f44d67a2fccd6dc20dde70%7C0%7C0%7C637980797572420627%7CUnknown%7CTWFpbGZsb3d8eyJWIjoiMC4wLjAwMDAiLCJQIjoiV2luMzIiLCJBTiI6Ik1haWwiLCJXVCI6Mn0%3D%7C3000%7C%7C%7C&sdata=9uW%2F1BtAZ0vv5WiFYcge8wA8TLD%2BKTUXi8fEqmTfaIw%3D&reserved=0)  **unacceptable-data-access-restrictions. Note that it is not acceptable for the authors to be the sole named individuals responsible for ensuring data access.**  **We will update your Data Availability statement to reflect the information you provide in your cover letter.** | | Following your remark, we will make all anonymized data of the focus group publicly available after acceptance of this article. The data will be available on DataverseNL and will be assigned with a digital object identifier (DOI). |  | |
|  | **Additional editor comments** | | | | |
| 5 | **In general, we would expect qualitative studies to include the following:**   1) **defined objectives or research questions;** 2) **description of the sampling strategy, including rationale for the recruitment method, participant inclusion/exclusion criteria and the number of participants recruited;** 3) **detailed reporting of the data collection procedures;** 4) **data analysis procedures described in sufficient detail to enable replication;** 5) **a discussion of potential sources of bias; and** 6) **a discussion of limitations. Please pay attention to these requirements when revising your manuscript.** | | We would like to thank the editor for providing us with the requirements for a qualitative study. Our study meets, the requirements mentioned by the editor. |  | |
| **Nr** | **Reviewers comment** | | **Authors’ response** | **Change in manuscript** | |
|  | **Comments** | | | | |
|  | **Reviewer #1** | |  |  | |
| 1 | **Introduction To long introduction, the authors must shorten it before publication.** | | We agree with the reviewer and, consequently, shortened the introduction.  The initial word count was: 1003, which is currently at: 627. | Pages 3-4. | |
| 2 | **Study design and data allocation**  **The authors state in lines 149-151 that it was not possible due to Covid-19 pandemic to assess physical measurements, it could be a bias in the selection of inclusion and exclusion criteria, it must be discussed as a limitation.** | | Agreed, changed in manuscript | Lines 696-701 below section ‘(strengths and) limitations’:  Additionally, due to the COVID-19-pandemic a neurological assessment was not performed to diagnose LOPS. Although subjects were not specially diagnosed with LOPS for this study, they were neurologically assessed by a physician who diagnosed persons with LOPS prior to this study. It was not possible to verify whether the subjects who were recruited via digital platforms and social media had indeed been diagnosed with LOPS by a physician. | |
| 3 | **LOPS: authors declare that patients with LOPS were included in the research, but auto perception of LOPS is not a valid method to diagnose DPN and LOPS, authors must explain it in depth and if no neurological assessment was performed, authors might explain how it can be done and if no possible add as a limitation in the discussion section accordingly.** | | Agreed, changed in manuscript | Lines 130-133:  Due to the COVID-19 pandemic, physical measurements were not possible, which is why the information on the LOPS (diagnosed by a physician in a neurological assessment prior to this study), foot deformities (diagnosed by a physician prior to this study), body weight, and height was self-reported.  Lines 696-701 below section ‘(strengths) and limitations’:  Additionally, due to the COVID-19-pandemic a neurological assessment was not performed to diagnose LOPS. Although subjects were not specially diagnosed with LOPS for this study, they were neurologically assessed by a physician who diagnosed persons with LOPS prior to this study. It was not possible to verify whether the subjects who were recruited via digital platforms and social media had indeed been diagnosed with LOPS by a physician. | |
| 4 | **Results Higher risk of ulcer occurrence patients seem to have higher knowledge about DFU complications derived from the results presented in table 2, despite this, the qualitative analyses of the results do not let us to reach any conclusion.** | | Following the remark, we made changes to table 2 to clarify the information provided in the table.  From (the revised) table 2 one could gather that all groups have sufficient knowledge on diabetes related complications. In fact, the most important complication (DFU) was mentioned by all groups. All groups also knew that therapeutic footwear is prescribed to prevent DFUs. However, the difference between the groups (based on the results of the qualitative study) is that it seems that one has to have experienced an ulcer before evaluating the factor ‘ulcer prevention’ important enough (top 3 in table 2) to start using therapeutic footwear (TF). This means that in table 2 the emphasis for the difference between group-noTF&noHoU, group-TF&noHoU and group-TF&HoU, is on the experience of an ulcer rather than on the risk for an ulcer occurrence.  The conclusion that is reached from this qualitative study is that it seems that patients have to experience an ulcer before to find ‘ulcer prevention’ important enough to start using TF. Even with experiencing an ulcer before, patients do not rank this factor the highest. This finding emphasizes that besides focusing on the factors that are perceived as important by different groups of persons with DM, one also has to find a way to educate (motivate) persons on the importance of using TF to prevent ulcers. | Table 2 is shown on page 10.  We summarized for all groups together what their knowledge was on the reasoning behind and function of TF. We also added the ranks to showcase the difference in importance of the factors per group (where you can see that ‘preventing ulceration’ is ranked the third place for the group that experienced ulcers before while the two other groups with no ulcer experience ranked that factor lower).  Lines 742-749 (conclusion):  Different experiences with TF as well as a HoU influence which factors are perceived as important for TF use. The factors comfort and fit and stability/balance were ranked in the top 3 of all groups. Taking these factors into account during the development and prescription process of TF may lead to increased adherence. The most interesting finding was that subjects only realized the importance of giving priority to ulcer prevention after experiencing an ulcer. Nevertheless, even with a HoU, subjects still did not rank this factor highly. This finding emphasizes that besides focusing on the factors that are perceived as important for TF use, person-centered education on the importance of using TF to prevent ulcers is also essential. | |
| 5 | **Also, the three studied groups were well balanced in terms of baseline characteristics. That said, the plausibility of the results may be limited by the relatively low number of participants. Was a sample size calculation performed? 8 patients per groups is very small, this need to be included as a limitation of the study.** | | We agree with the reviewer that for a quantitative study, 8 patients per group would have been too small. However, for a qualitative analysis a group of 8 people is a good size. We based our number of patients per group on literature about qualitative studies. Below you can find studies where it is stated that it is preferred/required to have a group of around 8 for a qualitative study in the form of a focus group.   - <https://www.semanticscholar.org/paper/Designing-and-Conducting-Focus-Group-Interviews-Krueger/eb7499b9e559c969d0a8e7bceafd36adc4578eaf> - <https://www.fhi360.org/sites/default/files/media/documents/Qualitative%20Research%20Methods%20-%20A%20Data%20Collector's%20Field%20Guide.pdf> - <https://pubmed.ncbi.nlm.nih.gov/29262162/> |  | |
| 6 | **The paper has a big weakness due to the lack of quantitative data, for further research, authors should confirm this results in a quantitative study.** | | We thank the reviewer to bring this point to our attention. We specifically chose a qualitative analysis of the focus group data since we were interested in understanding what factors patients find important (and their importance weight) in using therapeutic footwear and the reasoning (experience, attitude, wishes) behind the importance of the factors for the patients. Qualitative data (gathered from focus groups) on behavior, experience, and attitude can be difficult to quantify, with the possibility of losing the context and narrative behind the qualitative data. According to Tenny et al. one should not quantify data if they are not meant to be quantified. But indeed, we do think that next to a qualitative study (methods such as interviews and focus groups etc.), a quantitative study (based on a quantitative method such surveys and questionnaires etc.) will have an added value. Through a survey one could analyze whether a larger patient population agrees with the ranking of the different factors and if not, to rearrange the rank. One could also ask whether there are some missing factors etc. The study of Tenny et al. (see for the link below) emphasis the importance of both quantitative and qualitative studies and how they can be complementary to each other.   - <https://pubmed.ncbi.nlm.nih.gov/29262162/> | We added the following lines 676-681 to the discussion section:  A qualitative design highlights the reasoning (experiences, perceptions, and wishes) behind the importance attached to factors that influence the use of TF. A quantitative design could be the next step in analyzing whether a larger population agrees with the ranking of the different factors and also whether factors are missing. Tenny et al. advocate the use of different types of designs (qualitative and quantitative) because they provide complementary information.[35] | |
| 7 | **Discussion**  **The discussion is coherent, but I recommend shortening it.** | | We agree with the reviewer and shortened the discussion accordingly.  The initial word count was: 3194, which is currently at: 2470. | On pages 24-30, a considerable amount of lines were taken out. | |
|  | | **Reviewer #2** | | | |
| 8 | | **Even though you have made clear that ethics approval was not required, i feel that your work does require ethical approval for an international publication. Therefore can get retrospective approval?** | Thank you for this suggestion. It may be unclear that with a non-WMO approval we still had to follow the required legal acts and/or guidelines: the Medical Treatment Agreement (WGBO), General Data Protection Regulation (GDPR) and codes of conduct of the FEDERA (Federation of Medical Scientific Institutions). This is why we added the following line to the manuscript: ‘The required legal acts and/or guidelines, the Medical Treatment Agreement (WGBO), General Data Protection Regulation (GDPR) and codes of conduct of the FEDERA (Federation of Medical Scientific Institutions), were followed.’  We do not think a retrospective approval is needed for the following reasons:  The Medical Ethics Committee (METc) of the University Medical Center Groningen is legally authorized to evaluate research that is conducted by the UMCG. The METc decided our study is not subject to the Medical Research Involving Human Subjects Act (WMO). Research is subject to the WMO if the following criteria are met:   1. It concerns medical scientific research and 2. Participants are subject to procedures or are required to follow rules of behaviour.   Our participants were not subject to procedures or were required to follow rules of behaviour. This is why we agree with the decision of the METc that our study does not need a WMO approval. This does not mean that we did not follow other legal acts and/or guidelines. We followed the required legal acts and/or guidelines: the Medical Treatment Agreement (WGBO), General Data Protection Regulation (GDPR) and codes of conduct of the FEDERA (Federation of Medical Scientific Institutions). There has been also several international publications that did not require a WMO approval. Even studies published by PLOS ONE:   - [User-relevant factors determining prosthesis choice in persons with major unilateral upper limb defects: A meta-synthesis of qualitative literature and focus group results (europepmc.org)](https://europepmc.org/backend/ptpmcrender.fcgi?accid=PMC7326229&blobtype=pdf) - [Pre-operative rehabilitation for dysvascular lower-limb amputee patients: A focus group study involving medical professionals \| PLOS ONE](https://journals.plos.org/plosone/article?id=10.1371/journal.pone.0204726) | On lines 97-100 we added the following:  The required legal acts and/or guidelines, the Medical Treatment Agreement (WGBO), General Data Protection Regulation (GDPR) and, codes of conduct of the FEDERA (Federation of Medical Scientific Institutions) were followed. | |
| 9 | | **you have talked about the 'tool' of qualitative research, which I feel is not the right term to use. I also feel it would be nice for you to clarify that you are 'exploring new knowledge generation' which justifies this type of methodology.** | Agreed, changed in manuscript | The following was added in response to the reviewer’s comment on lines 80-85:  A qualitative study is a suitable means to generate new knowledge on factors that are perceived as important for the use of TF (in different groups of persons with DM and LOPS). This type of methodology is not limited by pre-specified questions and can therefore identify the most important factors as well as the underlying perceptions, experiences, and wishes of participants regarding use of TF.[14] Focus group discussions with room for interaction will likely result in a fruitful discussion where ideas emerge that provide in-depth insight into the topic.[15] | |
| 10 | | **some of your references in the introduction (4, 11,12,13) are quite old. Are there any more recent? if not state that this is a gap in the literature** | We agree with the reviewer and replaced the references. | Reference 11, 12, 13 were replaced by:  Waaijman R, Keukenkamp R, de Haart M, Polomski WP, Nollet F, Bus SA. Adherence to wearing prescription custom-made footwear in patients with diabetes at high risk for plantar foot ulceration. Diabetes Care. 2013;36(6):1613–8.  Arts MLJ, de Haart M, Bus SA, Bakker JPJ, Hacking HGA, Nollet F. Perceived usability and use of custom-made footwear in diabetic patients at high risk for foot ulceration. Journal of Rehabilitation Medicine. 2014;46(4):357–62.  Kossioris, A., Tentolouris, N., Kyriazopoulou, V., Loupa, C. V., Markou, G., Marakomichelakis, G. E., & Tyllianakis, M. (2017). Initial and continued adherence to wearing appropriate footwear in people with diabetic foot disease: results of a pilot study. Hellenic Journal of Nursing Science, 10(4), 21-28.  Reference 4 was published in 2020.  We think the reviewer meant reference 3 instead of 4. We replaced reference 3 with another paper :  Edmonds, M., Manu, C., & Vas, P. (2021). The current burden of diabetic foot disease. Journal of Clinical Orthopaedics and Trauma, 17, 88-93. | |
| 11 | | **line 64 - reword. patients who have previously suffered.....for a recurrence of ulceration** | We agree with the reviewer, however we removed line 64 to shorten the introduction. |  | |
| 12 | | **line 65 - can you use international guidelines? as well as national?** | Thank you for your suggestion. Line 65 was not phrased clearly, the national guidelines follow the ‘International working Group on the Diabetic Foot’ (IWGDF) guidelines. Line 65 is changed in the manuscript and we added the reference of the IWGDF guideline: [Guidelines on the prevention of foot ulcers in persons with diabetes (IWGDF 2019 update) (iwgdfguidelines.org)](https://iwgdfguidelines.org/wp-content/uploads/2020/11/Bus_et_al-2020-IWGDF-prevention-guideline.pdf) | We rephrased the original and added the correct reference on lines 62-64:  According to the International Working Group on the Diabetic Foot (IWGDF) guidelines on the prevention and management of the diabetic foot, persons with DM with a high risk of developing ulcers need to use therapeutic footwear (TF) in order to offload high plantar pressure.[7] | |
| 13 | | **After reading the introduction, i am not clear if you are trying to influence design of footwear or influence behaviour change to wear TF. Behavuior change is a different concept and area that you may want to differentiate in this section to make it clear to the reader your intentions.** | We agree and changed the introduction where we now describe more clearly that the goal of our study is to get an overview of factors that are perceived as important for the design of footwear and their importance weight in the use of therapeutic footwear in different patient groups. The end goal (which is outside the scope of this study) is that the results of our study will help in the design of footwear and the selection process of them which in turn may lead to an increase in the use of therapeutic footwear and prevent further deterioration of the patient’s foot condition (e.g. diabetic foot ulcers). | The changes in the introduction are given on lines 66-70, where the end goal of the study is briefly mentioned:  In order to improve adherence and prevent further deterioration of the condition of the foot, it is important to increase knowledge and awareness among persons with DM as well as to identify factors that are perceived as important for the use of TF.[11] This information can be used for the (future) development of TF and further improvement of the prescription process.  Lines 91-93 (no new lines but the goal of the study is described in these lines):  The aim of this study is to provide insight into the factors perceived as important (weight) by different groups of persons with DM and LOPS when using TF (based on severity of the disease and experience in using TF). | |
| 14 | | **line 145 - how did you measure TF use in the questionnaire?** | We agree with the reviewer that we should be more explicit. We therefor added a line that the information on the disease and TF use were gathered from the questionnaire through a self-report. | Lines 126-127:  Prior to the focus group discussions, a self-reporting questionnaire was sent to all (24) subjects to gain knowledge about their disease and TF use (number of days per week and hours per day). | |
| 15 | | **Results: Table 1 is slightly confusing; too many abbreviations, no legend, too many groups of data grouped** | Agreed, we changed manuscript accordingly | The revised Table 1 is seen on page 9, where we added a legend, split the data and tried to keep the abbreviations to a minimum. | |
| 16 | | **table 2 - the first part is a lot of repeated text for the three groups; could you introduce the ranking numbers as another column?** | Agreed, changed in manuscript | On page 10 the revised Table 2 is showcased where we summarized for all groups together what their knowledge was on the reasoning behind and function of TF, in order not to have a lot of repeated text. We also added another column where the ranking numbers are introduced. | |
| 17 | | **after reading the main findings, there appears a lot of repetition to each group within the text. This made it difficult to read and to compare and contrast amongst groups. You could condense much of this with a re-working of the presentation.** | We agree with the reviewer and shortened the result section by avoiding the repetition the reviewer refers to.  The initial word count was: 5593 (with the patient quotes), which is currently at: 4613 (including the patient quotes) | On page 8, 11-24 a considerable amount of lines were taken out to shorten the result section. | |
| 18 | | **your discussion similarly has lots of repetition as you have tried to seperate the groups and report on each. As above suggestion, try to condense and bring your findings and themes together** | We agree with the reviewer and shortened the discussion by avoiding the repetition the reviewer refers to.  The initial word count was: 3194, which is currently at: 2470. | On pages 24-30, a considerable amount of lines were taken out. | |
|  | | **Reviewer #3** | | | |
| 19 | | **Line 30 rather than “do not use therapeutic footwear sufficiently, rewrite as “do not use therapeutic footwear consistently, properly, regularly or as expected.** | Agreed, changed in manuscript | Line 27-28:  Persons with diabetes mellitus (DM) and loss of protective sensation (LOPS) due to peripheral neuropathy do not use their therapeutic footwear (TF) consistently. | |
| 20 | | **Line 37, “TF & HoU)” should come first to allow for the writing in full for (HoU) history of ulceration** | Agreed, changed in manuscript | Line 35-39:  Subjects (n =24) were divided into 3 focus groups based on disease severity: ulcer history (HoU) versus no ulcer history (no-HoU) and experience with TF (TF) versus no experience (no-TF). For each group of 8 subjects (TF&HoU; TF&no-HoU; no-TF&no-HoU), an online focus group discussion was organized to identify the most important influencing factors. | |
| 21 | | **Line 37: the content of the bracket should come after the word “discussions”.** | Agreed, changed in manuscript | Line 35-39:  A qualitative study was performed using focus group discussions. Subjects (n =24) were divided into 3 focus groups based on disease severity: ulcer history (HoU) versus no ulcer history (no-HoU) and experience with TF (TF) versus no experience (no-TF). For each group of 8 subjects (TF&HoU; TF&no-HoU; no-TF&no-HoU), an online focus group discussion was organized to identify the most important influencing factors. | |
| 22 | | **The presentation of the abstract has not sufficiently provided comprehensive details of the focus of the study which included giving detailed information about the factors that factors that influence or could influence the use of therapeutic footwear. The authors should list the factors based on the outcome of the said focus groups.** | We agree and changed the abstract where we listed the top 3 ranked factors of each group. We focused on the top 3 ranked factors due to the limited amount of words instructed by the journal. | Lines 42-45:  The factors comfort and fit and stability/balance were ranked in the top 3 of all groups. Usability was ranked in the top 3 of group-TF&noHoU and group-noTF&noHoU. Two other factors, reducing pain and preventing ulceration were ranked in the top 3 of group-TF&noHoU and group-TF&HoU, respectively. | |
| 23 | | **The use of personal pronoun should be avoided as much as possible in all aspect of the manuscript. Every instance of personal should be rephrased in 3rd person pronoun Eg: lines 31-32: “If we understand what factors could influence their use” Better rephrased as “If factors that influence their use are understood.” Instead of “There, we aimed to provide insight in the factors that……” Better rephrased as “Therefore, this study aimed to provide insight in the factors that ……”** | Agreed, changed in manuscript  The initial sentences of the manuscript (including the background section of the abstract) are changed by Sonja Hintzen, who thoroughly edited our manuscript for language usage, which is why not the exact sentences, that were given as example by the reviewer (lines 31-32), are seen in the background section of the abstract. However, throughout the whole manuscript we rephrased sentences with personal pronoun to 3^rd^ pronoun. | Lines 28-33 of the background section in the abstract:  TF is essential to prevent foot ulceration. In order to improve compliance in using TF, influencing factors need to be identified and analyzed. Persons with a history of foot ulceration may find different factors important compared with persons without ulceration or persons who have never used TF. Therefore, the objective of this study was to determine factors perceived as important for the use of TF by different groups of persons with DM and LOPS.  &  Throughout the whole manuscript we rephrased sentences with personal pronoun to 3^rd^ pronoun. | |
| 24 | | **Line 67: correct “hereby” to “Thereby”** | Agreed, changed in manuscript, however the initial sentence was taken out and rephrased by Sonja Hintzen, who edited our manuscript for language usage. | The initial line (65 -) 67 in the original manuscript is changed to the following in the revised manuscript, lines 62-65:  According to the International Working Group on the Diabetic Foot (IWGDF) guidelines on the prevention and management of the diabetic foot, persons with DM with a high risk of developing ulcers need to use therapeutic footwear (TF) in order to offload high plantar pressure.[7] The risk of developing DFUs can be reduced significantly by using offloading footwear for most of the day.[8,9] | |
| 25 | | **Line 81: “Two studies in this review” should be corrected as “Two studies in the review”** | Agreed, changed in manuscript. The initial sentence was taken out and rephrased by Sonja Hintzen, who edited our manuscript for language usage.  However we followed the reviewers comment to change ‘this’ review into ‘the’ review. | Lines 72-74:  Most studies in the review reported associations of different factors with adherence: foot deformity and minor amputation, perceived severity of the foot condition, TF appearance, body mass index, diabetes type, and age.[13] | |
| 26 | | **Line 92: “Currently, we are designing prototype shoes……” should be rephrased as “Currently, the authors of this study are designing prototype shoes …..”** | Thank you for the suggestion, however we removed line 92 to shorten the introduction. |  | |
| 27 | | **Lines 131-134: The groups described should be done properly by differentiated them using numerical number.** | In the manuscript the three groups were differentiated and shown in Figure 1. The reference to Figure 1 was not done properly (line 117 gave an error). We made sure the reference to the Figure is corrected. |  | |
| 28 | | **Detailed information as it relates to the number of participants recruited for the study on the basis of the recruitment channels should be presented and included in the study. That will possibly be of help to future researchers in the aspect of considering recruitment channel for related study design.** | The researchers of this study were not the main physicians treating the patients. Due to privacy reasons the researcher do not know exactly the number of patients recruited from each channel. |  | |
| 29 | | **How many participants were involved in the online questionnaire? This information should be provided.** | We agree and added this information in the manuscript. | We added this information in lines 126 (-127):  Prior to the focus group discussions, a self-reporting questionnaire was sent to all (24) subjects to gain knowledge about their disease and TF use (number of days per week and hours per day). | |
| 30 | | **Line 153-154: on how the discussion was organised online? Or “on how the discussion was to be organised” Please rewrite the sentence for clarity.** | Agreed, changed in manuscript | Lines 135-136:  Prior to the online meetings, all subjects received a letter with instructions on how the discussion was to be organized. | |
| 31 | | **Line: 155: Once all participants joined ….. Should be corrected as “When all participants joined ….”** | Agreed, changed in manuscript. The initial sentence was taken out and rephrased by Sonja Hintzen, who edited our manuscript for language usage.  However we followed the reviewers comment to change ‘Once..’ into ‘When..’. | Lines 137-139:  When joining the online meeting, subjects were informed (again) that the discussions were audio and video recorded and that they had to reaffirm consent. | |
| 32 | | **Line: 157; Correct “Hereafter” as “Thereafter”** | Agreed, changed in manuscript | Lines 139-140:  Thereafter, the moderator explained the purpose of the study, followed by a brief introduction of the subjects and the discussion round. | |
| 33 | | **Table 2: The word “patients” should be replaced with “persons” or “subjects”.** | Agreed, changed in manuscript.  In Table 2 we changed the word ‘patients’ to ‘subjects’ since ‘subjects’ is a term that is also used in the PLOS ONE journal guidelines. | Page 10: Table 2. We changed in the table ‘patients’ to ‘subjects’.  Throughout the whole manuscript we replaced the word ‘patient(s)’ with ‘person(s)/subjects(s)’. | |
| 34 | | **There is the need for the authors to include more references to support the discussion. The total number of cited references is too limited for an academic paper of this magnitude. The authors should search thoroughly as there are series of published articles in this area of research.**  **The authors may can consider using the following keywords: “Therapeutic footwear, Neuropathic Foot, Diabetes” to search for very current and relevant and updated literatures.  Articles published within the last three years (2019 to date) should form significant aspect in the study to enable the authors provide updated information in the field to the readers.** | The reviewer rightfully mentions the number of papers is too limited for the discussion sections. We therefor added a number of relevant references to the discussion section.  Not all the added references are from 2019-2022, however many studies related to the use of therapeutic footwear (and the factors that are related to therapeutic footwear) are older. Even a review from 2019 (see below for the link of the study) where the objective was, to identify in the literature aspects related to the recommendation of health professionals and the use of therapeutic footwear by patients with Diabetes Mellitus, the studies found and were interesting to our study were not very recent.  chrome-extension://dagcmkpagjlhakfdhnbomgmjdpkdklff/enhanced-reader.html?openApp&pdf=https%3A%2F%2Flink.springer.com%2Fcontent%2Fpdf%2F10.1007%2Fs40200-019-00428-9.pdf | The added references in the discussion section:   1. van Netten, J. J., Dijkstra, P. U., Geertzen, J. H., & Postema, K. (2012). What influences a patient’s decision to use custom-made orthopaedic shoes?. *BMC musculoskeletal disorders*, *13*(1), 1-7. 2. Coffey, L., Mahon, C., & Gallagher, P. (2019). Perceptions and experiences of diabetic foot ulceration and foot care in people with diabetes: A qualitative meta‐synthesis. *International wound journal*, *16*(1), 183-210. 3. Jarl, G., Tranberg, R., Johansson, U., Alnemo, J., & Lundqvist, L. O. (2020). Predictors of adherence to wearing therapeutic footwear among people with diabetes. *Journal of Foot and Ankle Research*, *13*(1), 1-9. 4. Albright, B. C., & Woodhull-Smith, W. M. (2009). Rocker bottom soles alter the postural response to backward translation during stance. *Gait & posture*, *30*(1), 45-49. 5. Waaijman, R., Keukenkamp, R., de Haart, M., Polomski, W. P., Nollet, F., & Bus, S. A. (2013). Adherence to wearing prescription custom-made footwear in patients with diabetes at high risk for plantar foot ulceration. *Diabetes care*, *36*(6), 1613-1618. 6. Williams, A. E., & Nester, C. J. (2006). Patient perceptions of stock footwear design features. *Prosthetics and orthotics international*, *30*(1), 61-71. 7. Arts, M. L., De Haart, M., Bus, S. A., Bakker, J. P., Hacking, H. G., & Nollet, F. (2013). Perceived usability and use of custom-made footwear in diabetic patients at high risk for foot ulceration. *Custom-made footwear in diabetes: Offloading, usability and ulcer recurrence*, 97. 8. Macfarlane, D. J., & Jensen, J. L. (2003). Factors in diabetic footwear compliance. *Journal of the American Podiatric Medical Association*, *93*(6), 485-491. 9. Tenny, S., Brannan, G. D., Brannan, J. M., & Sharts-Hopko, N. C. (2022). Qualitative Study. *StatPearls*. StatPearls Publishing. | |
|  | | **Reviewer #4** | | | |
| 35 | | **Page 3, Line 56, Please check the space between the reference and the following lines. Also, the spacing is inconsistent in other areas, such as Page 3, line 63 and other references on this page and also throughout the article. Please make them all consistent.** | Agreed, changed in manuscript | | Throughout the whole manuscript we tried to correct the spacing. |
| 36 | | **Page 4, Line 101. Please check grammar for “proofed”.** | We agree, however we removed Line 101 in order to shorten the introduction. | |  |
| 37 | | **Page 5, Line 123. Please clarify if the Orthopedic shoe technicians are titled as “Pedorthists” in the Netherlands. If so, please use the appropriate professional title and please check other studies from the Netherlands have been using the term.**  **Ref:** [**https://onlinelibrary.wiley.com/doi/full/10.1002/dmrr.3237**](https://onlinelibrary.wiley.com/doi/full/10.1002/dmrr.3237) | Agreed, changed in manuscript | | Throughout the whole manuscript we changed ‘shoe technician’ in ‘pedorthists’. |
| 38 | | **Page 6, Line 150, Please clarify if there was an option to collect or verify the patient data such as LOPS, and foot deformities from a verified medical record source or referral source other than the patient’s self-report on them.** | We added a number of lines to clarify how the information on the LOPS was gathered. | | Lines 130-133:  Due to the COVID-19 pandemic, physical measurements were not possible, which is why the information on the LOPS (diagnosed by a physician in a neurological assessment prior to this study), foot deformities (diagnosed by a physician prior to this study), body weight, and height was self-reported.  We also added the following lines below the section ‘(strength and) limitations’, lines 696-702:  Additionally, due to the COVID-19-pandemic a neurological assessment was not performed to diagnose LOPS. Although subjects were not specially diagnosed with LOPS for this study, they were neurologically assessed by a physician who diagnosed persons with LOPS prior to this study. It was not possible to verify whether the subjects who were recruited via digital platforms and social media had indeed been diagnosed with LOPS by a physician. However, there was no reason for the subjects to be dishonest about their diagnosis since they did not benefit from participating in the study. |
| 39 | | **Page 11, Line 242. Please be consistent with using Capital for “ Appearance” and “prescription process”.** | Agreed, changed in manuscript | | We changed the original line 242 and added the following on lines 233-234:  For example, the factors appearance and prescription process are also ranked in Table 2.  &  Throughout the whole manuscript we tried to be consistent with using capital/non-capital for ‘appearance’ and ‘prescription process’ |
