# Supplementary material for: Factors influencing the use of therapeutic footwear in persons with diabetes mellitus and loss of protective sensation: A focus group study
Source: PLoS One. 2023 Jan 12;18(1):e0280264. doi: 10.1371/journal.pone.0280264 (PMC9836263; doi:10.1371/journal.pone.0280264)
Supplement: Attachment — Submitted filename: Response to Reviewers.docx [file pone.0280264.s010.docx]

Dear Editor,

We are glad to hear our manuscript is acceptable for publication. We would like to thank you and the reviewers for your time and critical view on the article. We have answered the fifth reviewer’s question/comment below and reported which changes were made accordingly in the manuscript.

|  | **Journal requirements** | | |
| --- | --- | --- | --- |
| **Nr** | **Editor comments** | **Authors’ response** | **Change in manuscript** |
|  | **Comments** | | |
| 5 | **Journal requirements:**  **Please review your reference list to ensure that it is complete and correct. If you have cited papers that have been retracted, please include the rationale for doing so in the manuscript text, or remove these references and replace them with relevant current references. Any changes to the reference list should be mentioned in the rebuttal letter that accompanies your revised manuscript. If you need to cite a retracted article, indicate the article’s retracted status in the References list and also include a citation and full reference for the retraction notice.** | We would like to thank the editor for providing us with the journal requirements for the reference list. We reviewed our reference list, which is complete and correct. |  |
| **Nr** | **Reviewers comment** | **Authors’ response** | **Change in manuscript** |
|  | **Comments** | | |
|  | **Reviewer #5** |  |  |
| 1 | **Adherence to treatment has been considered as an important factor to influence those clinical outcomes of RCTs. Patients who were adherent had significantly better outcomes than those who were nonadherent. This research plays an important role in discovering new information. The authors have further improved the manuscript according to reviewers’ comments and also explained every question reasonably. However, the following question require further clarification: 1.The exclusion criteria are too simple. Many comorbidities such as cardiac insufficiency, severe hepatic and renal dysfunction, malignant tumor, unsteady gait, lower extremity edema, et.al may influence the choice of the main reason regarding low adherence to use of TF. The characteristics of the subjects should show more information of comorbidities and complications.** | We would like to thank the reviewer to bring this point to our attention. In order to provide more information on the comorbidities and complications, we made changes to Table 1. The most frequent comorbidities in the three focus groups were shown in Table 1. The less frequent comorbidities (extended comorbidities list), are summarized as ‘other’ in Table 1. However, for the interested readers, we also provided another table (S1 Table; table in the ‘supporting information’), with each of the less frequent comorbidity listed per focus group and not summarized as ‘other’. | The revised Table 1 is seen on line 210, where we added the information on the (most frequent) comorbidities per focus group.  The new S1 Table is added as a ‘Supporting information’ file where the extended comorbidities per focus group are shown.  In the Manuscript, lines 192-193 & 852 refer to the supporting information table (S1 Table) |
